# Evaluating the effects of material properties of artificial meniscal implant in the human knee joint using finite element analysis

**DOI:** 10.1038/s41598-017-06271-3

**Published:** 2017-07-20

**Authors:** Duraisamy Shriram, Gideon Praveen Kumar, Fangsen Cui, Yee Han Dave Lee, Karupppasamy Subburaj

**Affiliations:** 1 Engineering Product Development (EPD) Pillar, Singapore University of Technology and Design (SUTD), 8 Somapah Road, Singapore, 487372 Singapore; 2 Institute of High Performance Computing, A*STAR, Singapore, 138632 Singapore; 3 Department of Orthopaedic Surgery, Changi General Hospital, Singapore, 529889 Singapore

## Abstract

Artificial meniscal implants may replace severely injured meniscus and restore the normal functionality of the knee joint. Implant material stiffness and shape influence the longevity of implantations. This study, using 3D finite element analysis, aimed to evaluate the effects of material stiffness variations of anatomically shaped artificial meniscal implant in the knee joint. Finite element simulations were conducted on five different cases including intact knee, medial meniscectomized knee, and the knee joint with the meniscal implant with three distinct material stiffness. Cartilage contact pressures, compression stresses, shear stresses, and implant kinematics (medial-lateral and posterior-anterior displacement) were evaluated for an axial compressive load of 1150 N at full extension. Compared to the meniscectomized knee, the knee joint with the meniscal implant induced lower peak cartilage contact pressure and reduced the cartilage regions loaded with contact pressures greater than the peak cartilage contact pressure induced by the intact knee. Results of the current study also demonstrate that cartilage contact pressures and implant displacement are sensitive to the implant material stiffness. The meniscal implant with a stiffness of 11 MPa restores the intact knee contact mechanics, thereby reducing the risk of physiological damage to the articular cartilage.

## Introduction

The menisci play a crucial role in the knee, by stabilizing the joint and disseminating forces across the articulating surfaces^1^. Consequently, the upkeep of the native intact menisci is vital for healthy functioning of the knee. Meniscal tears are the most widely known knee injuries, with a rate of meniscus related injuries leading to meniscectomy (partial or total removal of meniscus) of 61 per 100,000 populace for every year^2^. The meniscal tears observed in acute knee injuries cause hyperextension or hyperflexion of the knee in younger patients or act as a risk factor for degeneration in the elderly. Medial meniscal tears occur more often than lateral meniscus tears, at a quantitative relation of approximately 2:1^3^.

It is understood that the total meniscectomy prompts progressive articular cartilage wear following a couple of years after surgery. The key fact behind this is the alteration in the global biomechanics of the knee and thereby to increase the articular instability, leading to a progressive and degenerative arthroscopic pathology^4, 5^. The patients with symptomatic osteoarthritis (OA) might be treated by meniscal allograft transplantation (i.e. supplanting of the local tissue with a benefactor meniscus) to alleviate pain and restore knee function^6^. Meniscal allografts were seen to shrivel and experience collagen remodeling upon transplantation^7, 8^, which affects the mechanical strength and may lead to allograft tears, articular instability, and degenerative damage. Moreover, issues concerning the provision and size of allograft, the risk of disease transfer, rejection, infection, and high expenses^7, 8^ have driven the need for alternate treatment options. Recently, studies have shown that a functional synthetic full meniscal implant could conceivably overcome the deficiencies of meniscus allograft transplantation^9–12^.

The improved designs of meniscal implants were extensively tested in the animal models, leading to severe damage to the articular cartilage caused by large deformation of the meniscal implant and wear-particle deposition^13, 14^. A synthetic implant design resembling the native meniscus as close as possible may provide a successful long-term implantation and restore the normal joint function^9, 15^. However, considerable attention has not been given to design and development of anatomically shaped meniscal implants and its effect on the overall biomechanics of the knee joint.

Since the primary function of the meniscus is to resist and disseminate the forces across the articulating surfaces, the mechanical properties of the meniscal implants play a major role in their function^16, 17^. Isotropic materials with different mechanical properties, such as material stiffness, are being used to manufacture the implants. However, the required material stiffness for optimal functionality of isotropic meniscal implants is not yet reported in the literature. It is challenging to experimentally study the effect of material stiffness of the meniscal implant on the knee joint contact mechanics after implantation. Computational simulation tools like finite element (FE) simulation can be employed to overcome this challenge. FE simulation is the widely used computational tool to estimate stresses within intact, morbid, injured and implanted knee joints^18–23^. Thus, it is a viable tool in providing physiological insights and assessing critical design parameters of the meniscal implants.

Measuring the magnitude and monitoring the distribution of contact pressures on the articular cartilage after medial meniscectomy or implantation have been viewed as a reliable approach for evaluating the biomechanical behavior in disseminating the loads across the articulating surfaces^9, 18–23^. Therefore, the primary objectives of the current study were to (1) compare the articular cartilage contact pressures and their distributions and meniscus/implant displacement in intact knee, medial meniscectomy (total removal of medial meniscus) and meniscal implant and (2) study the influence of meniscal implant material stiffness variations on the articular cartilage contact pressures and implant displacement, using FE simulations on a subject-specific knee joint.

## Materials and Methods

### Methodology

**Figure 1:**
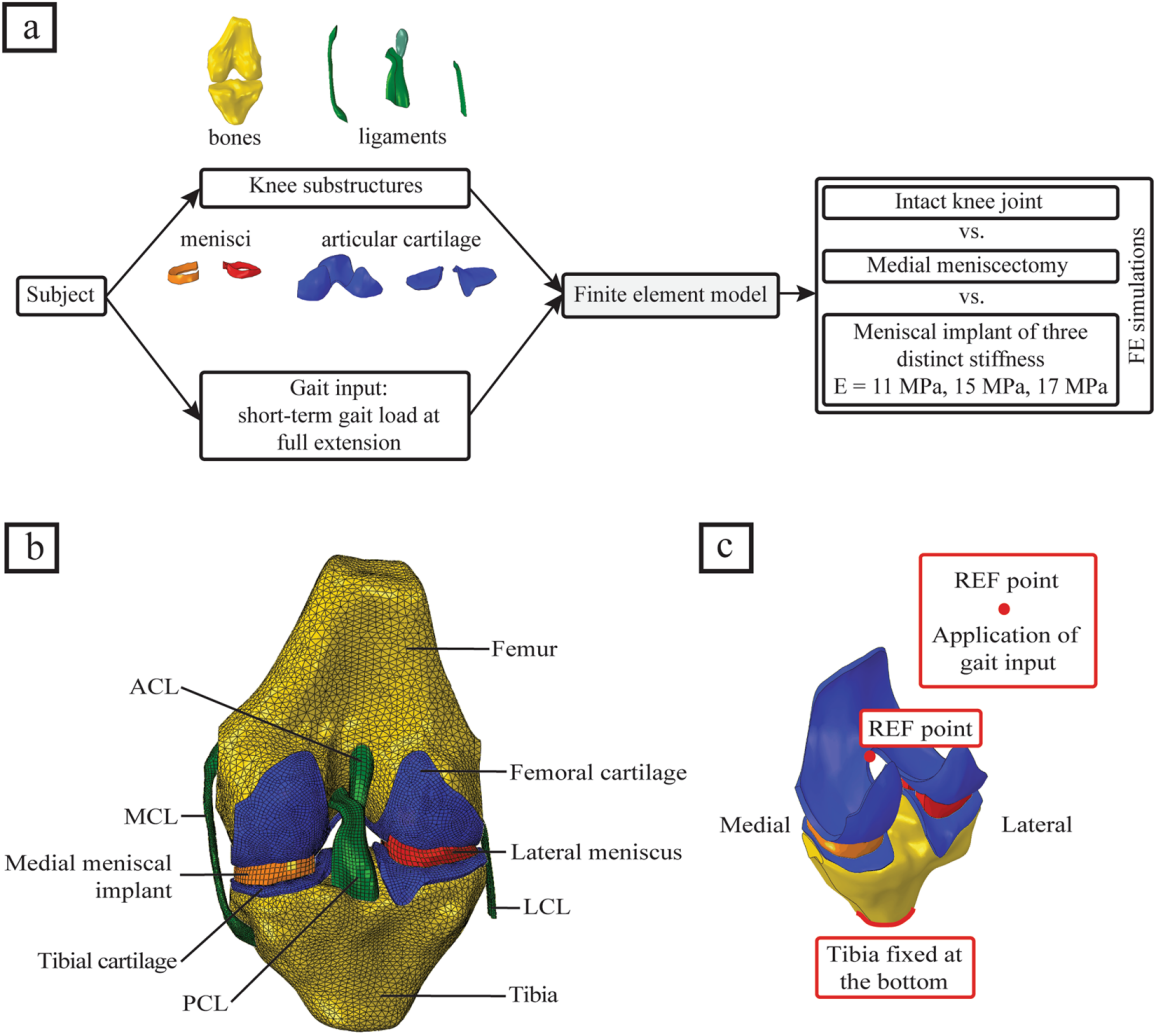
(**a**) Study workflow, (**b**) 3D finite element model of the human knee joint (posterior view), including the bones, the articular cartilages, the ligaments, the lateral meniscus and the medial meniscal implant, and (**c**) model boundary conditions showing the reference point where the short term gait input is applied.

All methods were carried out in accordance with relevant guidelines and regulations. A schematic representation of the proposed methodology, to study cartilage contact pressures and their distributions in the human knee joint with intact knee, medial meniscectomy and with the meniscal implant, is shown in Fig. 1a. Adapting the geometries of knee substructures from the Open Knee project^24, 25^, a 3D FE model of the human knee joint was developed. At first, FE simulations were conducted on the knee joint model with the intact medial meniscus. Then, the simulations were carried out on the knee joint model with medial meniscectomy. At last, the native medial meniscus was replaced by the anatomically shaped arbitrary meniscal implant, and the simulations were repeated for the same with three distinct material stiffnesses. The contact pressures, compression stresses, and shear stresses on the articular cartilage were evaluated for each meniscal case studied. Additionally, for the intact meniscus and implant cases, the kinematics of medial meniscus or implant including medial-lateral and anterior-posterior translations were evaluated.

### FE model of the knee joint

The geometry of the right knee substructures, extracted from MR (magnetic resonance) images, of a cadaveric knee specimen (70 years old, female) was obtained from the Open Knee project^24, 25^. The 3D knee substructures were imported into Abaqus ver. 6.14-2 (SIMULIA, Providence, RI, USA) and a finite element model of the human knee joint was developed (Fig. 1b). The bone models (femur and tibia) were meshed using three-noded 3D rigid triangular facet elements (Abaqus R3D3); the soft tissues (cartilage, ligaments, and menisci) and anatomically shaped arbitrary meniscal implant were meshed using eight-noded hexahedral elements (Abaqus C3D8). The ligament-bone and cartilage-bone attachment were modeled by attaching the nodes on the relating surfaces. The contact surfaces of cartilage-cartilage and cartilage-menisci were simulated using frictionless sliding contact elements for both the lateral and medial sections. Assuming the ligaments extracted from MR images were in extended position, a pre-strain of 4% was applied to the lateral collateral ligament (LCL) and medial collateral ligament (MCL) and 5% was applied to the anterior cruciate ligament (ACL) and posterior cruciate ligament (PCL)^26^. The meniscal horn attachments were simulated using linear springs with no pre-strain and the combined spring stiffness of 350 Nmm^−1^ on each side^27^ and attached to the top of the tibia.

### Material models

Using a user-defined UMAT in Abaqus CAE ver. 6.14-2, the native menisci and the ligaments were modeled as transversely isotropic hyperelastic neo-Hookean materials^25, 28–31^ with the strain-energy function:

1 $${\rm{\Phi }}={C}_{10}(\overline{{G}_{1}}-3)+\frac{1}{{D}_{1}}{({J}_{F}-1)}^{2}+S(\lambda )$$

where C_10_ denotes the bulk material constant associated with the shear modulus µ (C_10_ = 2/µ), *J*_*F*_ denotes the Jacobian of the deformation gradient **F**, and $\overline{{G}_{1}}$ represents the first invariant of the left Cauchy-Green tensor $\overline{{G}_{1}}=tr\overline{{\bf{F}}}{\overline{{\bf{F}}}}^{T}$ with the modified deformation gradient $\overline{{\bf{F}}}$ $(\overline{{\bf{F}}}={{J}_{F}}^{-0.33}{\bf{F}})$. Here, S(λ) denotes the strain energy function of the fiber family which satisfies the conditions:

2 $$\lambda \frac{dS}{d\lambda }=\{\begin{array}{ll}0, & \lambda \le 1\\ {C}_{3}(\exp ((\lambda -1){C}_{4})-1), & 1 < \lambda  < {\lambda }^{\ast }\\ {C}_{5}\lambda +{C}_{6}, & \lambda \ge {\lambda }^{\ast }\end{array}$$

The fiber stretch, λ, denotes the strain in these fibers and determined from the deformed fiber orientation **a**_**d**_, the deformation gradient **F**, and the initial fiber orientation **a**_**0**_ $(\lambda \cdot {{\bf{a}}}_{{\bf{d}}}={\bf{F}}\cdot {{\bf{a}}}_{{\bf{0}}})$. These fibers would not support compressive stresses if they were under compression λ ≤ 1. The stiffness of these fibers increases exponentially if the fiber stretch is greater than one but less than a pre-defined value, λ^*^ (Table 1). Beyond this value, these fibers get straighten and the stiffness increases linearly. The initial fiber orientation **a**_**0**_ was specified according to the geometry of the local element. In the ligaments, the fibers were oriented in line to the principal axis of the geometry. In the meniscus, the fibers were oriented circumferentially to resist hoop stresses while loading^32, 33^. The constants C_3_, C_4_, and C_5_ denote the rate of exponential stress, the rate of collagen uncrimping, and Young’s modulus of the straightened fibers, respectively. The constant C_6_ symbolizes the stress continuation at λ^*^ $[{C}_{6}=(\exp ({C}_{4}({\lambda }^{\ast }-1))-1)\cdot {C}_{3}-({C}_{5}\cdot {\lambda }^{\ast })]$. The material constants C_10_, C_3_, C_4_, C_5_, and D_1_, are listed in Table 1.

**Table 1:** Material constants for the ligaments (ACL: Anterior Cruciate Ligament; PCL: Posterior Cruciate Ligament; MCL: Medial Collateral Ligament; LCL: Lateral Collateral Ligament) and the intact meniscus^24, 29^.

Soft Tissue		C_10_ (MPa)	C_3_ (MPa)	C_4_ (−)	C_5_ (MPa)	D_1_ (MPa^−1^)	${\lambda }^{\ast }$ (−)
Ligaments	ACL	1.95	0.0139	116.22	535.039	0.01366	1.046
	PCL	3.25	0.1196	87.178	431.063	0.0082	1.035
	MCL	1.44	0.57	48.0	467.1	0.00252	1.063
	LCL	1.44	0.57	48.0	467.1	0.00252	1.063
Meniscus		4.61	0.1197	150.0	400.0	0.01085	1.019

The bony structures (femur and tibia) were modeled as rigid bodies as the stiffness of the bone is several orders of magnitude higher than that of the cartilages^29, 31^. The articular cartilages were modeled as isotropic single-phase linear elastic material with an elastic modulus (E) of 10 MPa and a Poisson’s ratio (ν) of 0.45^34, 35^. The sensitivity analysis of cartilage contact stresses and contact area to the changes in elastic modulus of the articular cartilage was perfomed (refer Supplementary Tables S1–S3). The material for meniscal implant (Bionate^®^ grade II polycarbonate urethane, types 80 A, 90 A, and 55D, DSM Biomedical, Pennsylvania, USA) was modeled as an isotropic neo-Hookean material with a Poisson’s ratio ν = 0.49. Three distinct material stiffness of the meniscal implant (E = 11 MPa, 15 MPa, and 17 MPa) were considered to simulate all the three types of Bionate^®^ grade II polycarbonate urethane material.

### Boundary and loading conditions

The bottom of the tibial bone was fixed in all translational and rotational degrees of freedom, while the femur was only fixed in knee flexion where it is fixed at 0° to simulate a short-term gait load of a human knee joint in full extension. A reference point, located in the central region between lateral and medial femoral epicondyles, was coupled to the femoral surface using the constraint method in Abaqus (Fig. 1c). An axial compressive load of 1150 N was applied to the femoral condyle reference point, which relates to the gait cycle load in full extension (0° flexion angle)^21, 36^.

### Mesh convergence

After employing material modeling, boundary conditions and loading conditions on the intact knee model, sensitivity analyses on mesh density were performed to verify that the model predictions were not affected by mesh refinement. The element size of knee substructures was varied to yield six different mesh resolutions, by keeping the very refined mesh as the reference for comparison (Table 2). The peak joint contact pressure predicted by cases a-e were compared with those predicted by the reference case, and the cases within ±5% of the reference case were considered as accurate. Case c was found to be optimal, as it requires less computing power while maintaining prediction accuracy of 96% with respect to the reference case model. The predictions by cases d and e were very inaccurate (>5%). The human knee joint finite element model (Fig. 1b) included 3D structures of the femur (2 mm element size, 10510 elements), tibia (2 mm element size, 7985 elements), femoral articular cartilage (1 mm element size, 13948 elements), medial tibial articular cartilage (1 mm element size, 2794 elements), lateral tibial articular cartilage (1 mm element size, 3298 elements), medial meniscal implant (1 mm element size, 1512 elements), lateral meniscus (1 mm element size, 1984 elements), ACL (1 mm element size, 1107 elements), PCL (1 mm element size, 1840 elements), MCL (1 mm element size, 12820 elements), and LCL (1 mm element size, 1953 elements).

**Table 2:** Sensitivity analyses on mesh density for different knee substructures.

Case (s)	Element size (ES) and number of elements (NOE)								% change in peak cartilage contact pressure
	Femoral cartilage		Tibial cartilage		Medial meniscus		Lateral meniscus		
	ES (mm)	NOE	ES (mm)	NOE	ES (mm)	NOE	ES (mm)	NOE	
Reference	0.75	37155	0.5	42423	0.5	9630	0.5	14640	—
Case a	0.75	37155	0.5	42423	1	1512	1	1984	1.93
Case b	0.75	37155	1	6092	1	1512	1	1984	2.78
Case c	1	13948	1	6092	1	1512	1	1984	3.97
Case d	1.5	3252	1.5	1358	1.5	444	1.5	516	8.43
Case e	2	1944	2	845	2	224	2	272	13.27

## Results

### Comparison of articular cartilage contact pressures and meniscus/implant displacement in intact knee, medial meniscectomized knee, and the knee joint with the meniscal implant

**Figure 2:**
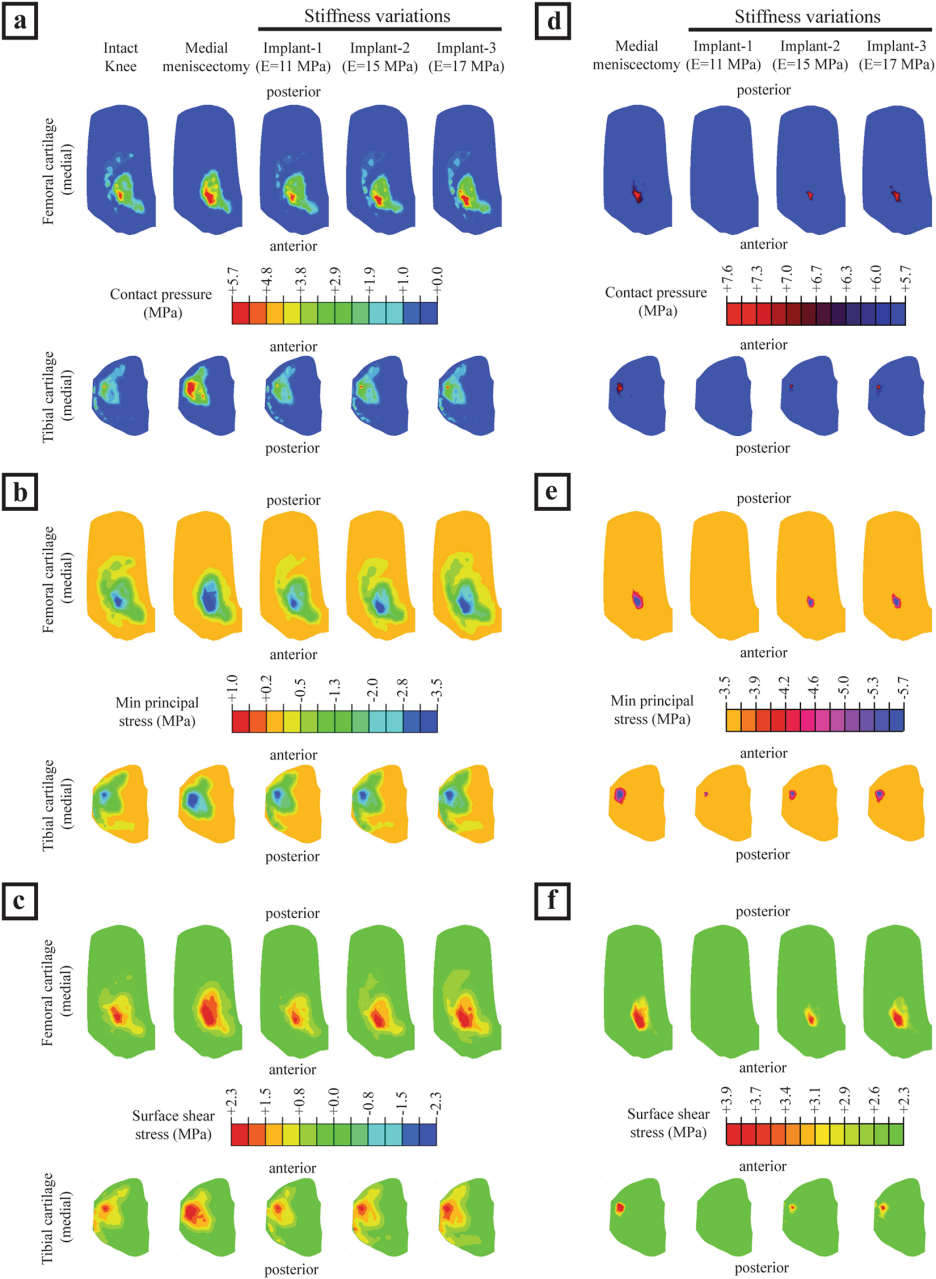
(**a–c**) The medial cartilage contact pressures, compression stresses (minimum principal) and shear stresses on the femoral and tibial surfaces for the intact knee, medial meniscectomized knee and the knee joint with the meniscal implant with three distinct material stiffness, and (**d–f**) femoral and tibial surfaces with contact pressures, compression stresses and shear stresses higher than the peak values of those induced by the intact knee.

**Figure 3:**
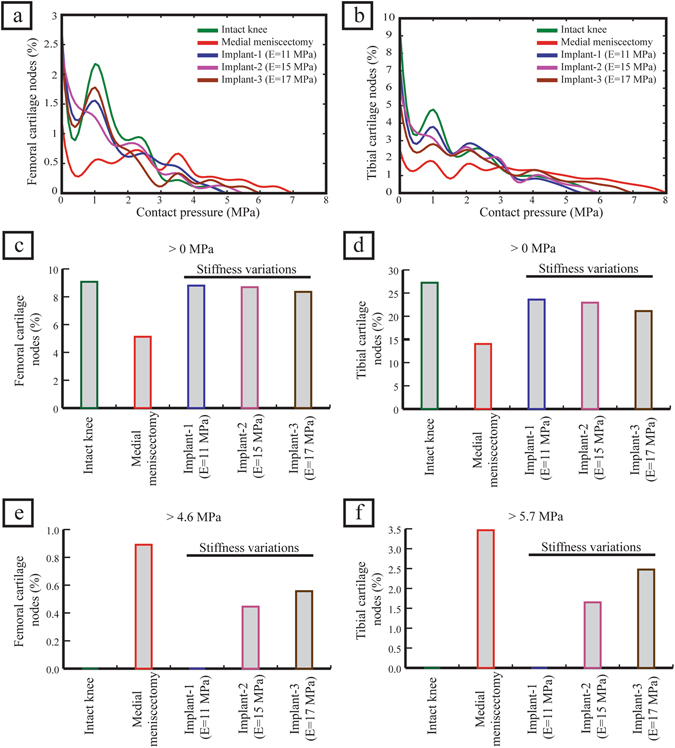
Analysis of articular cartilage contact pressure. (**a,b**) the cartilage contact pressure for each node (only non-zero values were included), (**c,d**) the percentage of cartilage nodes loaded with contact pressure greater than 0 MPa (reflects the loaded cartilage area), (**e**) the percentage of femoral cartilage nodes loaded with contact pressure greater than 4.6 MPa (peak femoral cartilage contact pressure induced by the intact knee), and (**f**) the percentage of tibial cartilage nodes loaded with contact pressure greater than 5.7 MPa (peak tibial cartilage contact pressure induced by the intact knee).

**Figure 4:**
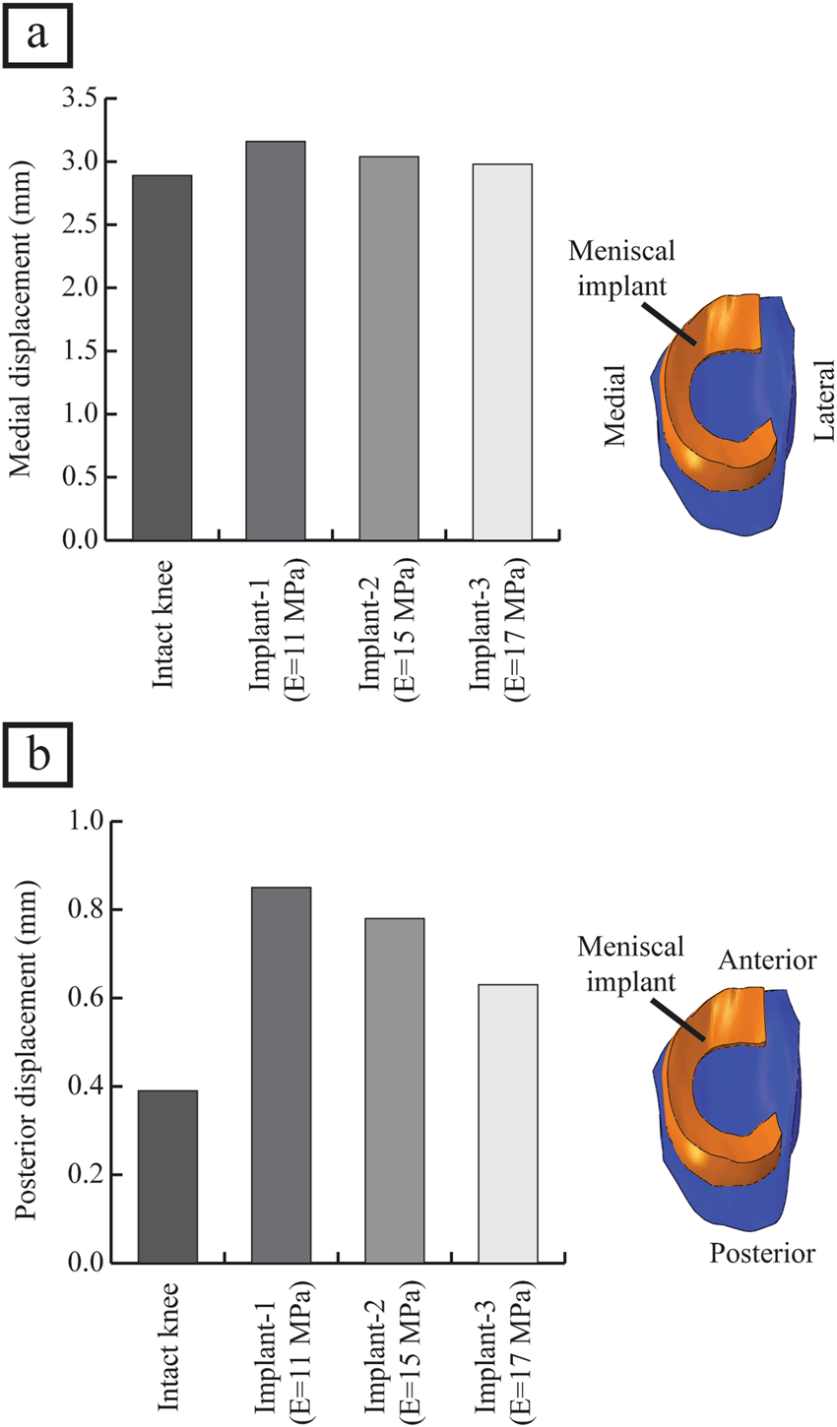
(**a**) The medial displacement of the meniscus or implant induced by the intact knee and the knee joint with the meniscal implant with three distinct material stiffness, and (**b**) the posterior displacement of the meniscus or implant induced by the intact knee and the knee joint with the meniscal implant with three distinct material stiffness, loaded with an axial compressive joint load of 1150 N.

The intact knee joint disseminated the contact pressures, compression stresses and shear stresses over a large area of femoral and tibial cartilage. The medial meniscectomized knee reduced the cartilage contact area which resulted in higher values of contact pressures focused on the central region of cartilage. The meniscal implant with a stiffness of 11 MPa (hereafter referred to as implant-1) redisseminated the pressures by loading larger surface area covering central and peripheral regions of the cartilage resulting reduced peak cartilage pressure compared with medial meniscectomy (Fig. 2a). In the knee joint with implant-1, the femoral and tibial cartilages were not loaded with contact pressures greater than 4.6 MPa (peak femoral cartilage pressure induced by the intact knee) and 5.7 MPa (peak tibial cartilage pressure induced by the intact knee), respectively, while in the medial meniscectomized joint, the articular cartilages experienced significantly higher contact pressures (>4.6 MPa for femoral cartilage and >5.7 MPa for tibial cartilage) (Figs 2d, 3a,b,e,f). The medial meniscectomized joint resulted in reduced total loaded area compared to the intact knee and implant-1 on the femoral and tibial cartilages due to the focus of cartilage pressures on the central region of the tibial cartilage (Fig. 3c,d). The maximum contact pressure induced on the articular cartilages by the knee joint with implant-1 was considerably lower than the medial meniscectomized joint and proximate to the intact knee (4.6 MPa vs. 6.7 MPa vs. 4.6 MPa on the medial femoral cartilage and 5.2 MPa vs. 7.6 MPa vs 5.7 MPa on the medial tibial cartilage). The cartilage compression stresses (minimum principal), shear stresses and cartilage compressive strains induced by implant-1 follows the same trend as cartilage contact pressures (Fig. 2b,c,e,f) (Table 3). The medial meniscus/implant displacement induced by the knee joint with implant-1 were larger than those of the intact knee (medial direction: 3.2 mm vs. 2.9 mm; posterior direction: 0.9 mm vs. 0.4 mm) (Fig. 4a,b).

**Table 3:** Comparison of the peak cartilage contact pressure, the peak cartilage compressive stress, the peak cartilage shear stress, and the peak cartilage compressive strain induced on the articular cartilage of the models with intact meniscus, medial meniscectomy and the meniscal implant with three distinct stiffness.

		Cases				
		Intact knee	Medial Meniscectomy	Meniscal implant-1 (E = 11 MPa)	Meniscal implant-2 (E = 15 MPa)	Meniscal implant-3 (E = 17 MPa)
Peak cartilage contact pressure (MPa)	Femoral	4.64	6.65	4.56	4.98	5.51
	Tibial	5.74	7.63	5.15	5.97	6.61
Peak cartilage compressive stress (MPa)	Femoral	2.76	4.47	2.66	3.91	4.06
	Tibial	3.52	5.67	3.63	5.01	5.39
Peak cartilage shear stress (MPa)	Femoral	1.93	3.53	1.81	2.21	2.89
	Tibial	2.32	3.92	2.29	2.85	3.26
Peak cartilage compressive strain (%)	Femoral	11.9	15.7	11.6	12.4	13.3
	Tibial	13.8	16.8	13.8	14.7	15.4

### Influence of meniscal implant stiffness variations on the articular cartilage contact pressures and implant displacement

The meniscal implant with variations in material stiffness affected the contact pressures on the articular cartilage and implant displacement. Compared to the knee joint with implant-1, the meniscal implant with stiffness of 15 MPa (hereafter referred to as implant-2), and the implant with a stiffness of 17 MPa (hereafter referred to as implant-3), increased the area of cartilage region loaded with contact pressures greater than 4.6 MPa (peak femoral cartilage pressure induced by the intact knee) and 5.7 MPa (peak tibial cartilage pressure induced by the intact knee) on the femoral side and tibial side, respectively (Figs 2a,d, 3a,b,e,f). Implant-3 resulted in decreased total loaded cartilage area compared to implant-1 and implant-2 on the femoral and tibial cartilage (Fig. 3c,d). The peak contact pressures induced by the knee joint with implant-3 were higher than implant-2 and implant-1 (5.5 MPa vs. 5.0 MPa vs. 4.6 MPa on the medial femoral cartilage and 6.6 MPa vs. 6.0 MPa vs 5.2 MPa on the medial tibial cartilage). The compression stresses (minimum principal), shear stresses and cartilage compressive strains induced on the articular cartilages by the knee joint with implant-3 follows a similar trend as cartilage contact pressures (Fig. 2b,c,e,f) (Table 3). The implant displacement induced by implant-3 were lower than those of implant-2 and implant-1 (medial direction: 3.0 mm vs. 3.1 mm vs. 3.2 mm; posterior direction: 0.6 mm vs. 0.8 mm vs. 0.9 mm). (Fig. 4a,b). Hence, an increase in implant material stiffness increased the cartilage contact pressures, compression stresses, shear stresses and compressive strains, on the other hand, reduced the implant displacement.

## Discussion

Supplanting a severely injured meniscus with an anatomically-shaped artificial implant is becoming a promising treatment procedure for patients suffering from severe meniscal injuries. Given the advancements in medical imaging, design, and manufacturing technologies, an anatomically-shaped implant geometry could be reproduced to fit the intricate articular surface of the distal femur and the proximal tibia. The optimal choice of the stiffness of material for meniscal implant plays a crucial role in improving contact pressures experienced by the knee joint after medial meniscectomy. In this study, we have carried out FE simulations to investigate the effects of meniscal implant material stiffness on articular cartilage contact pressures and implant kinematics. The critical findings of the current study were: (1) the peak cartilage contact pressure induced by the knee joint with implant-1 was lower compared to the medial meniscectomy and proximate to the intact knee, (2) the variations in meniscal implant material stiffness affected the cartilage contact pressures and implant displacement, and (3) increasing the material stiffness of the meniscal implant increased the cartilage contact pressures, but reduced the implant displacement.

When compared with the medial meniscectomy, implant-1 reduced the peak contact pressure, peak compressive stress and peak shear stress by 31%, 41%, and 49% on the medial femoral cartilage and by 33%, 18% and 42% on the medial tibial cartilage, respectively. When compared with the intact knee, the knee joint with implant-1 increased the displacement in the medial and posterior directions by 9% and 54%, respectively. The increase in implant displacement may be due to the lack of circumferential reinforcement in these synthetic implants. Despite such large displacement, the peak contact pressure induced on the articular cartilage by the knee joint with implant-1 was proximate to the peak contact pressure observed in the intact knee joint.

Leatherman *et al*. studied the sensitivity of cartilage contact stresses to the shape and material properties of the meniscus. The study found that the shape of the meniscus had a smaller effect on contact stresses when compared to the material properties of the meniscus^37^. In the current study, by increasing the stiffness of the isotropic meniscal implant, there was a significant change in the contact mechanics of the knee joint. Implant-1 induced lower peak contact pressure, compression stress and shear stress on the articular cartilage compared to implant-2 and implant-3. The meniscal implant with high stiffness (implant-3) showed less displacement but induced high magnitude of peak contact pressure on the articular cartilage. Higher magnitude contact pressures on the articular cartilage may respond adversely causing biological changes in the cartilage and the underlying bone which may lead to early OA^17^.

The intact meniscal shape and horn attachments are regenerated by designing an anatomically shaped meniscal implant, which is necessary for the proper functioning of the meniscus by stabilizing the human knee joint and disseminating the load across the articulating surfaces^38, 39^. Contrasted with commercial meniscal implants like NUsurface^®^, which do not have an intact horn attachment like setup^9, 40^, an anatomically shaped meniscal implant considered in this study functions efficiently after transplantation.

Some studies in the literature have performed FE simulations to predict the magnitude and distribution of contact pressures and compression stresses on the medial tibial cartilage in the intact knee and medial meniscectomized knee^21, 41–44^. Peña *et al*. considered an axial compressive loading of 1150 N at full extension and reported the peak compression stress of 3.36 MPa on the medial tibial cartilage for the intact knee. In medial meniscectomized knee, the peak compression stress on the medial tibial cartilage was increased up to 5.34 MPa^21^. Similar outcomes have been obtained from our FE simulations, i.e. a peak compressive stress of 3.52 MPa on the medial tibial cartilage for the intact knee and a peak compressive stress of 5.67 MPa on the medial tibial cartilage in the case of the meniscectomized knee. Donahue *et al*. considered an axial compressive load of 800 N at full extension and reported the mean contact pressure of 2.35 MPa on the medial tibial cartilage for the intact knee^43^, while we predicted a mean contact pressure of 3.09 MPa on the medial tibial cartilage for the intact knee under 1150 N. Meng *et al*. predicted the contact area on the medial tibial plateau for the intact knee. Under an axial compressive load of 1000 N at full extension, the estimated medial tibial plateau contact area was 614 mm^2^ ^44^. Our FE simulation for the intact knee predicted the contact area of 684 mm^2^ on the medial tibial plateau under 1150 N, in line with the reported values in the literature.

Wilson *et al*. developed an axisymmetric model to simulate the meniscectomized knee^45^. They predicted a 40% decrease of contact area on the medial tibial plateau for the meniscectomized knee compared to the intact knee^45^. In our study, we predicted a 48% decrease of contact area on the medial tibial plateau, which corresponds to published research findings^21, 46, 47^. The meniscectomized knee has undesirable effects, as evident from our FE simulations (Table 3). In the meniscectomized knee, the peak contact pressure, peak compression stress, and peak shear stress increases about 35%, 61%, and 70%, respectively, compared to the intact knee. The medial articular cartilage nodes were loaded with high contact stresses in the meniscectomized knee, while those regions were either not loaded or loaded with low values of contact stresses in the intact knee (Figs 2d–f, 3e,f). The change of contact conditions implies instability and therefore affects the salubrious functioning of the knee joint^21^.

The estimated peak contact pressure on the medial tibial cartilage from our FE simulation for the intact knee is in good agreement with experimentally measured contact pressure reported by Verma *et al*.^46^ and Allaire *et al*.^48^. At 1000 N axial compressive load, the experimentally measured peak contact pressures after medial meniscectomy were ranging from 6.0 MPa to 11.3 MPa^46–48^, while the peak contact pressures were ranging from 8.3 MPa to 12.4 MPa at 1800 N axial compressive load^49, 50^. The peak contact pressure from our FE simulation at an intermediate axial compressive loading of 1150 N after medial meniscectomy is in good agreement with these studies available in the literature. There are numerous challenges in experimentally measuring the contact stresses with a meniscal implant, for instance, the pressure sensor or film insertion may change the biomechanical contact conditions of the knee joint^51^. Besides, it is not possible to experimentally measure shear stresses on the articular cartilage surface as it may cause severe damages to the outermost of the articular cartilage strata. In the current study, FE simulations were utilized to investigate the considered cases efficiently. In order to extrapolate the outcomes of FE simulation to the *in-vivo* scenario, it is essential to validate the simulation results by comparing with the experimentally measured data. For this study, we have used the Open Knee substructures for developing the FE model, so the cadaveric knee specimen was not available for biomechanical experiments. However, we have compared our FE simulation results with the experimentally measured data available in the literature to check the accuracy of results predicted by our FE model. To give a reference to the cases that we have considered, the peak contact pressure on the medial tibial cartilage induced by the intact knee and the medial meniscectomized knee from our study were compared with experimental investigations in the literature (Table 4). By considering the results derived from the FE simulations of intact knee and medial meniscectomized knee as references for comparing a knee with the meniscal implant, our results could well explain the functioning of the meniscal implant and this information can be used to compare with germane *in-vivo* clinical cases.

**Table 4:** Comparison of the peak tibial cartilage contact pressure in the current study and the experimentally measured values available in the literature.

Experimental/computational study		Flexion angle (°)	Axial compressive load (N)	Peak tibial cartilage contact pressure (MPa)		% increase in peak cartilage contact pressure
				Intact Knee	Medial Meniscectomy	
Experimental	Verma *et al*.^46^	0	1000	6.0	11.3	47
	Fukubayashi *et al*.^47^	0	1000	3.0	6.0	50
	Allaire *et al*.^48^	0	1000	5.0	6.4	22
	Lee *et al*.^49^	0	1800	4.5	12.4	64
	Paletta *et al*.^50^	0	1800	2.4	8.3	71
Current study (computational)		0	1150	5.7	7.6	25

Several confinements ought to be considered while deciphering the outcomes of our study. The FE model was built using the geometric information from single subject data, so probably another model might respond differently to the loading conditions applied in this study. However, our FE model was developed from a cadaveric knee specimen of the average-sized female subject, and thus the outcomes from our study are applicable to patients with average-sized knee joints with no anatomical deformities. In the current study, standard static simulations were carried out on only one knee geometry with simple loading and boundary conditions. Also, the outcomes of the meniscal implant FE simulation were compared with intact knee joint FE simulation where the native meniscus is simulated which may not have some characteristics of the real meniscus tissue. Cartilages were modeled as linear elastic and isotropic material, however, incorporating computationally expensive biphasic material behavior would be more precise^21, 29^. When subjected to short loading times, the fluid flow is not substantial and hence the response of elastic material does not deviate from the biphasic material^43^. The instantaneous response of cartilages to a short-term gait load was focused in the current study, therefore cartilages can be modeled as elastic materials. Also, viscoelastic and swelling material properties were not considered while modeling the menisci and the articular cartilage^45^. In future, dynamic FE simulations will be performed for the complete gait cycle loading and boundary conditions to better predict the biomechanics of the knee joint, with possible extension to also study the biomechanical effects of size and shape variations of the meniscal implant.

In summary, we have demonstrated that an anatomically shaped meniscal implant prevents higher magnitude contact pressures on articular cartilage when compared with medial meniscectomy using FE simulations. The contact pressures on the articular cartilages and the implant displacement are sensitive to the material stiffness of polycarbonate urethane meniscal implant. At 1150 N axial compressive load, the peak contact pressure on the articular cartilage induced by the meniscal implant modeled from a neo-Hookean material with a stiffness of 11 MPa was proximate to those induced by the native intact meniscus. Increasing the material stiffness of the meniscal implant led to elevated contact pressures on the articular cartilage which increases the risk of physiological damage to the articular cartilage, however, reduced the implant displacement. These critical findings will be utilized to optimize the required mechanical characteristics of the anatomically shaped meniscal implants and eventually accomplish successful meniscal transplantation in a clinical scenario.

## Electronic supplementary material

Supplementary Tables S1-S3
