# Supplementary material for: Evaluating the effects of material properties of artificial meniscal implant in the human knee joint using finite element analysis
Source: Sci Rep. 2017 Jul 20;7:6011. doi: 10.1038/s41598-017-06271-3 (PMC5519683; doi:10.1038/s41598-017-06271-3)
Supplement: Supplementary file 1 — Supplementary Tables S1-S3 [file 41598_2017_6271_MOESM1_ESM.doc]

**Supplementary Information**

**Evaluating the effects of material properties of artificial meniscal implant in the human knee joint using finite element analysis**

Duraisamy Shriram^1^, Gideon Praveen Kumar^2^, Fangsen Cui^2^, Yee Han Dave Lee^3^, Karupppasamy Subburaj^1*^

^1^ Engineering Product Development (EPD) Pillar, Singapore University of Technology and Design (SUTD), 8 Somapah Road, Singapore 487372

^2^ Institute of High Performance Computing, A*STAR, Singapore 138632

^3^ Department of Orthopaedic Surgery, Changi General Hospital, Singapore 529889

*Corresponding author: Subburaj Karupppasamy, [subburaj@sutd.edu.sg](mailto:subburaj@sutd.edu.sg)

**Supplementary Table S1.** Sensitivity analysis of cartilage peak contact pressure to the changes in elastic modulus of the articular cartilage

| Applied axial compressive load = 1000 N | | | | | | | |
| --- | --- | --- | --- | --- | --- | --- | --- |
| Elastic modulus of cartilage  (MPa) | E = 5 | E = 8 | E= 10 | E = 12 | E = 15 | E = 18 | E = 20 |
| Medial tibial cartilage  peak contact pressure  (MPa) | 5.3 | 5.4 | 5.7 | 6.0 | 6.3 | 6.3 | 6.4 |
| Experimentally measured value = 5.96 MPa under 1000 N load (Verma *et al*. 2008) | | | | | | | |
| % difference in peak contact pressure | 13.5 | 10.5 | 4.6 | 0.1 | 5.2 | 5.5 | 7.0 |

Note: The range of variation of elastic modulus was based on the published literature ^21,29,31,51^

**Supplementary Table S2.** Sensitivity analysis of cartilage contact area to the changes in elastic modulus of the articular cartilage

| Applied axial compressive load = 1000 N | | | | | | | |
| --- | --- | --- | --- | --- | --- | --- | --- |
| Elastic modulus of cartilage  (MPa) | E = 5 | E = 8 | E= 10 | E = 12 | E = 15 | E = 18 | E = 20 |
| Medial tibial cartilage  contact area  (mm2) | 709 | 687 | 643 | 625 | 568 | 530 | 515 |
| Experimentally measured value = 612 mm^2^ under 1000 N load (Verma *et al*. 2008) | | | | | | | |
| % difference in contact area | 13.6 | 10.9 | 4.9 | 2.1 | 7.7 | 15.4 | 18.9 |

Note: The range of variation of elastic modulus was based on the published literature ^21,29,31,51^

**Supplementary Table S3.** Sensitivity analysis of cartilage peak compressive stress to the changes in elastic modulus of the articular cartilage

| Applied axial compressive load = 1150 N | | | | | | | |
| --- | --- | --- | --- | --- | --- | --- | --- |
| Elastic modulus of cartilage  (MPa) | E = 5 | E = 8 | E= 10 | E = 12 | E = 15 | E = 18 | E = 20 |
| Medial tibial cartilage  peak compressive stress  (MPa) | 3.4 | 3.5 | 3.5 | 3.8 | 4.0 | 4.2 | 4.2 |
| Numerically predicted value = 3.36 MPa under 1150 N load (Peña *et al*. 2005) | | | | | | | |
| % difference in peak compressive stress | 0.1 | 4.6 | 4.7 | 12.1 | 16.1 | 19.1 | 19.6 |

Note: The range of variation of elastic modulus was based on the published literature ^21,29,31,51^
